# Supplementary material for: The Systems Biology Research Tool: evolvable open-source software
Source: BMC Syst Biol. 2008 Jun 29;2:55. doi: 10.1186/1752-0509-2-55 (PMC2446383; doi:10.1186/1752-0509-2-55)
Supplement: Additional file 1 — SBRT Archive. An archive of the current version of the Systems Biology Research Tool. [file 1752-0509-2-55-S1.zip › sbrt-1.4.0/doc/developers_guide/api/sbrt/shell/text/math/MatrixElementFormatV1.html]

MatrixElementFormatV1


|  |  |  |
| --- | --- | --- |
| **Overview** \| **Package** \| **Class** \| **Use** \| **Tree** \| **Deprecated** \| **Index** \| **Help** | |  |
| **PREV CLASS**   **NEXT CLASS** | **FRAMES**    **NO FRAMES**     **All Classes** |  |
| SUMMARY: NESTED \| FIELD \| CONSTR \| METHOD | DETAIL: FIELD \| CONSTR \| METHOD |  |


---


## sbrt.shell.text.math Class MatrixElementFormatV1<R,C>

```
java.lang.Object
  sbrt.shell.text.math.MatrixElementFormatV1<R,C>
```

**Type Parameters:**: `R` - the row index type.: `C` - the column index type.

**All Implemented Interfaces:**: Format, Formatter<MatrixElement<R,C,java.lang.Double>>, MatrixElementFormat<R,C,java.lang.Double>, Parser<MatrixElement<R,C,java.lang.Double>>, SimpleFormat<MatrixElement<R,C,java.lang.Double>>

---

``` public final class MatrixElementFormatV1<R,C> extends java.lang.Object implements MatrixElementFormat<R,C,java.lang.Double> ```

This class is a concrete implementation of `MatrixElementFormat`.
The string representation of matrix elements used by this class is:
`[RowIndex | ColumnIndex] = Element`.

**Author:**
:   This class was written and documented by
    Jeremiah Wright while in the Wagner lab.

---

**Constructor Summary**

`MatrixElementFormatV1(SimpleFormat<R> rowIndexFormat,
SimpleFormat<C> columnIndexFormat)`
  
          Constructs a new matrix element format.


| **Method Summary** | |
| --- | --- |
| `<S extends MatrixElement<R,C,java.lang.Double>> java.lang.String` | `format(S indexPair)`             Returns a formatted string representation of the provided matrix element. |
| `java.lang.String` | `formatElement(MatrixElement<? extends R,? extends C,java.lang.Double> element)`             Returns a formatted string representation of the provided matrix element. |
| `DoubleFormatV1` | `getElementFormat()`             Returns the format used for matrix elements. |
| `IndexPairFormatV1<R,C>` | `getIndexFormat()`             Returns the format used for matrix elements. |
| `MatrixElement<R,C,java.lang.Double>` | `parse(java.lang.String element)`             Parses the provided string and returns its corresponding matrix element. |

**Methods inherited from class java.lang.Object**

`clone, equals, finalize, getClass, hashCode, notify, notifyAll, toString, wait, wait, wait`

**Constructor Detail**

### MatrixElementFormatV1

```
public MatrixElementFormatV1(SimpleFormat<R> rowIndexFormat,
                             SimpleFormat<C> columnIndexFormat)
```

:   Constructs a new matrix element format.

    **Parameters:**: `rowIndexFormat` - the row index formatter.: `columnIndexFormat` - the column index formatter.


**Method Detail**

### getIndexFormat

```
public IndexPairFormatV1<R,C> getIndexFormat()
```

:   Returns the format used for matrix elements.

    :   **Returns:**: the format used for matrix elements.

---


### getElementFormat

```
public DoubleFormatV1 getElementFormat()
```

:   Returns the format used for matrix elements.

    :   **Returns:**: the format used for matrix elements.

---


### formatElement

```
public java.lang.String formatElement(MatrixElement<? extends R,? extends C,java.lang.Double> element)
```

:   Returns a formatted string representation of the
    provided matrix element.

    :   **Parameters:**: `element` - the matrix element to be formatted. **Returns:**: a formatted string representation of the provided matrix element.

---


### format

```
public <S extends MatrixElement<R,C,java.lang.Double>> java.lang.String format(S indexPair)
```

:   Returns a formatted string representation of the
    provided matrix element.

    :   **Specified by:**: `format` in interface `Formatter<MatrixElement<R,C,java.lang.Double>>`
    :   **Type Parameters:**: `S` - the type of matrix element. **Parameters:**: `indexPair` - the matrix element to be formatted. **Returns:**: a formatted string representation of the provided matrix element.

---


### parse

```
public MatrixElement<R,C,java.lang.Double> parse(java.lang.String element)
```

:   Parses the provided string and returns its
    corresponding matrix element.

    :   **Specified by:**: `parse` in interface `Parser<MatrixElement<R,C,java.lang.Double>>`
    :   **Parameters:**: `element` - the string to be parsed. **Returns:**: the matrix element corresponding to the provided string.


---


|  |  |  |
| --- | --- | --- |
| **Overview** \| **Package** \| **Class** \| **Use** \| **Tree** \| **Deprecated** \| **Index** \| **Help** | |  |
| **PREV CLASS**   **NEXT CLASS** | **FRAMES**    **NO FRAMES**     **All Classes** |  |
| SUMMARY: NESTED \| FIELD \| CONSTR \| METHOD | DETAIL: FIELD \| CONSTR \| METHOD |  |


---
